# Are women with preterm labour at risk for negative birth experience? a comparative cross-sectional study from Iran

**DOI:** 10.1186/s12884-023-05575-9

**Published:** 2023-04-13

**Authors:** Zahra Najafi, Mojgan Mirghafourvand, Solmaz Ghanbari-Homaie

**Affiliations:** 1 Students Research Committee, Tabriz University of Medical Sciences, Tabriz, Iran; 2 Social Determinants of Health Research Center, Tabriz University of Medical Sciences, Tabriz, Iran; 3 Department of Midwifery, Faculty of Nursing and Midwifery, Tabriz University of Medical Sciences, Tabriz, Iran

**Keywords:** Premature birth, Patient Satisfaction, Birth satisfaction, Iran

## Abstract

**Background:**

The unpredictable nature of preterm labour can be a stressful experience for the mother. The occurrence of preterm birth can lead to the failure of the mother's previous expectations regarding the process of labour and birth leading to negative perception towards birth.

**Methods:**

This descriptive-analytical cross-sectional study was conducted in Tabriz, Iran. We employed convenience sampling to recruit eligible mothers with term birth (314 women) and preterm birth (157 women). Childbirth Experience Questionnaire 2.0, Preterm Birth Experiences and Satisfaction Scale, and Delivery Fear Scale were used to measure the woman’s fear of delivery during labour and birth experience. Data were analysed by general linear model.

**Results:**

The prevalence of negative birth experience in the term and preterm birth groups was 31.8% and 14.3%, respectively. The results of the multivariable general linear model, after the adjustment of demographic and obstetric characteristics, showed that there was no statistically significant difference between the two groups of mothers with term and preterm birth [β (95% CI): -0.06 (-0.22 to 0.09); *p* = 0.414] in terms of childbirth experience. However, the fear of delivery had a significant relationship with the childbirth experience [-0.02 (-0.03 to -0.01); *p* < 0.001].

**Conclusion:**

There was no statistically significant difference in terms of women’s childbirth experience between the mothers with term and preterm births. The fear of delivery during labour was the predictor of childbirth experience. In order to improve women's childbirth experience, interventions should be made to reduce their fear during labour.

## Background

Birth is one of the most important events in a woman's life. Women's emotional and cognitive experience of birth has a significant effect on the physical and mental state of the mother during the postpartum period [1] and her interaction with the infant [2]. For this reason, in recent decades, the views, expectations and experience of women have been given special attention [3].

The World Health Organization (WHO) defines the positive birth experience as follows: “one that fulfils or exceeds a woman’s prior personal and sociocultural beliefs and expectations” [4]. Usually, women with positive birth experience feel powerful and confident throughout their lives [5, 6]. However some women remember their birth as a negative experience in life. The prevalence of negative birth experience ranges from 8.6% to 44% [7]. The negative birth experience increases the risk of negative health outcomes such as postpartum depression [1] and fear of the next birth [8]. It can also lead to the request for cesarean delivery in the next birth [9, 10] and even the lack of desire for subsequent reproduction [11, 12]. Maternal age, parity, fear of childbirth, self-efficacy, participation, control, expectations, preparation, support, unexpected medical complications, analgesia during labour, and admission of the baby to Neonatal Intensive Care Unit (NICU) have been reported as the related factors with birth experience [7].

Gestational age and preterm labour have also been raised as other factors in association with birth experience [13]. The prevalence of preterm birth in the world is 11% [14] and in Iran it is 10% [15]. The unpredictable nature of preterm labour and the possibility of the baby being admitted to the NICU can be a stressful experience for the mother [16, 17]. The occurrence of preterm birth may belie the mother's previous expectations regarding the process of labour and birth, thus leading to negative perceptions towards birth [18]. Mothers following preterm birth have been reported to express distress and post-traumatic stress symptoms several years after birth [19, 20]. However, a group of mothers after preterm birth express positive experience of their birth and have a favorable attachment to the infant [21, 22]. There have actually been certain studies reporting a lower risk level for this group of women, due to the shorter duration of labour and fewer interventions [23].

Most studies have focused on the birth experience of low-risk and full-term women. Whereas women with preterm infants may experience their birth differently from women with term infants. Considering the importance of having a positive birth experience as one of the important indicators of the quality of care [24], the high prevalence of negative birth experience in Iran [25] and the prevention of negative birth experience according to the experience of each group, the study is aimed to compare the birth experience of women with preterm and term births.

## Methods

### Study design

This is a descriptive-analytical cross-sectional study that was conducted between March and August, 2022.

### Participants

Women with term birth (gestational age between 37 and 42 full weeks) and preterm birth (gestational age between 26 and 36 weeks and 6 days) were included in the study. Eligibility criteria included: residence in Tabriz city and suburban; healthy newborn; not having a history of depression based on the mother's self-report or medical record; not taking anti-depressants and not having a stressful event in life during the last three months prior to the study.

### Recruitment

Sampling method was convenient. Women admitted to the labour ward of Al-Zahra, Taleghani and 29 Bahman hospitals in Tabriz, Iran were examined in terms of eligibility criteria based on the checklist. After making sure of the eligible criteria, the objectives and method of conducting the study were explained to the women and written informed consent was obtained from them. The participants were assured that their information would remain confidential and anonymous. To complete the sampling of both groups at the same time, women were selected every day in the ratio of two term births to one preterm birth. In this way, first the preterm birth was identified and then simultaneously or immediately after this, the two term births were included in the study. Demographic and obstetric checklists were completed during labour and postpartum by reviewing medical records. Also, the fear of delivery questionnaire during labour and childbirth experience questionnaires within 24 h postpartum were completed by the interviewer.

### Setting

The participants from Al-Zahra educational, referral and specialized hospital, Taleghani general and educational hospital, and 29 Bahman organizational hospital of East Azerbaijan, Tabriz, Iran were included in the study. All three of these hospitals are level three hospitals.

### Data collection tools

Data collection tools in this study included demographic and obstetric checklists, Childbirth Experience Questionnaire (CEQ 2.0), Preterm Birth Experiences and Satisfaction Scale (P-BESS) and Delivery Fear Scale (DFS).

### Childbirth Experience Questionnaire 2.0 (CEQ 2.0)

The questionnaire contains 23 items, including the following subscales: “Own capacity”, “Professional support”, “Perceived safety” and “Participation”. The responses range from completely agree (score 4), to relatively agree (score 3), relatively disagree (score 2) and completely disagree (score 1). A higher score in the CEQ 2.0 indicates a more positive birth experience [26]. The CEQ 2.0 has been psychometrically tested in Iran. The score ≤ 2.5 has been considered as negative birth experience [27]. The CEQ 2.0 was used in order to compare birth experience in the same way in two preterm and term groups. The CEQ 2.0 was completed within 24 h postpartum.

### Preterm Birth Experiences and Satisfaction Scale (P-BESS)

This questionnaire has 17 questions within three subscales: [1] “Staff professionalism and empathy” (questions 2, 3, 4, 6, 7, 9 and 17), “Information and explanations” (questions 1, 5, 8, 11, 13, 15 and 16) and “Confidence in staff” (questions 10, 12 and 14). The questions are graded on a five-point Likert scale (1 = strongly disagree, 2 = disagree, 3 = no opinion, 4 = agree, 5 = strongly agree). Items 10, 12, 14 and 16 are scored in reverse. A higher score indicates more satisfaction with care during birth [28, 29]. The P-BESS has been psychometrically tested in Iran [23]. This specific tool was also used in our study in order to evaluate birth experience in the preterm group more accurately, in addition to CEQ 2.0. The P-BESS was completed within 24 h postpartum.

### Delivery Fear Scale (DFS)

The DFS includes 10 questions, with the scores ranging from 1 (I do not agree at all) to 10 (I completely agree). DFS was completed at the beginning of the active phase of labour [30]. The Persian version of this questionnaire was psychometrically tested and its Cronbach’s alpha coefficient was reported as 0.77 [31].

### Sample size

The sample size of 147 women was calculated based on the study of Najjarzadeh et al. [23] and considering of mean (standard deviation) = 75.6 (16.2), α = 0.05 and 90% power. Finally, considering the probability of 10% drop, the final sample size was calculated to be 157 women in the preterm group. The sample size of 283 women was calculated based on the study of Ghanbari et al. [25] and considering of mean (standard deviation) = 2.9 (0.92), α = 0.05 and 90% power. Finally, considering the probability of 10% drop, the final sample size was calculated to be 314 women in the term group.

### Ethical considerations

The study has been approved by the Ethics Committee of Tabriz University of Medical Sciences, Tabriz, Iran (Code: IR.TBZMED.REC.1400.1200). Written informed consent was obtained from all participants. In order to keep the information confidential, the coding method of the questionnaire was used without mentioning the name and surname. In the case of the participants who were illiterate, the informed consent form was explained by the researcher in their own language and their fingerprints were taken**.**

### Data analysis

Data were analyzed, using Version 24.0 software for Windows (IBM Inc., Armonk, NY, USA). Qualitative data were reported as frequency (percentage) and quantitative data as mean (SD: standard deviation). In univariate analysis, demographic and obstetric characteristics between two preterm and term groups using Independent-Samples T Test; Chi-square for trend; Fisher's Exact; Chi-square and Mann–Whitney U were compared. In the next step, the variables that were significant (*p* < 0.05) were entered into the general linear model, and by adjusting the effect of these variables, the birth experience was compared between the two groups.

## Results

About 420 women with term birth and 182 women with preterm birth who were willing to participate in the study were examined in terms of eligibility criteria. Finally, a total of 314 women with term birth and 157 women with preterm birth were included in the study. The mean (SD) age of the women in preterm and term groups was 28.0 (7.1) and 27.2 (6.4) years, respectively. There was a statistically significant difference between the two groups in terms of education and occupation (*p* < 0.05) (Table 1). The mean (SD) gestational age in the preterm and term groups was 34.5 (2.4) and 39.1 (0.9) weeks, respectively. There was also a statistically significant difference between the two groups in terms of parity, history of preterm labour, complications during pregnancy, duration of stay in the labour room, augmentation, use of pain relief method, skin to skin contact, NICU admission, baby weight and fear of birth (*p* < 0.05) (Table 2).

**Table 1:** Socio-demographic characteristics of the mothers by preterm and term labour

Characteristics	Preterm (*n* = 157)	Term (*n* = 314)	*p*
**Maternal age (years), Mean (SD)**	28.0 (7.1)	27.2 (6.4)	0.222^a^
**Husband age (years), Mean (SD)**	33.5 (6.2)	32.7 (5.7)	0.168^a^
**Maternal Educational level**			** < 0.001**^**b**^
Illiterate or elementary	69 (43.9)	85 (27.0)	
Secondary or high school	6 (3.8)	121 (38.5)	
Diploma	56 (35.7)	79 (25.2)	
Academic	26 (16.6)	29 (9.2)	
**Husband Educational level**			**0.004**^**b**^
Illiterate or elementary	24 (15.3)	71 (22.6)	
Secondary or high school	59 (37.6)	128 (40.7)	
Diploma	46 (29.3)	87 (27.7)	
Academic	28 (17.8)	28 (8.9)	
**Maternal Occupation,** Housewife	151 (96.2)	314 (100)	**0.001**^**c**^
**Husband Occupation**			**0.030**^**d**^
Employee	8 (5.1)	11 (3.5)	
Labor	11 (7.0)	48 (15.3)	
Self-employed	138 (87.9)	255 (81.2)	
**Life satisfaction**			0.579^b^
Not at all	0	1 (0.3)	
Relatively	43 (27.4)	75 (23.9)	
Completely	114 (72.6)	238 (75.8)	
**Income adequacy**			0.052^b^
Inadequate	12 (7.6)	43 (13.7)	
Relatively adequate	132 (84.1)	252 (80.3)	
Completely adequate	13 (8.3)	19 (6.1)	

The data indicate frequency (percent). *P* < 0.05 indicates significance difference

^a^Independent-Samples T Test

^b^Chi-square for trend

^c^Fisher’s Exact

^d^Chi-square

**Table 2:** Obstetric characteristics of the mothers by preterm and term labour

Characteristics	Preterm (*n* = 157)	Term (*n* = 314)	*p*
**Gestational age (Weeks), Mean (SD)**	34.5 (2.4)	39.1 (0.9)	< 0.001^a^
**Parity**			< 0.001^b^
1	70 (44.6)	90 (28.7)	
2	45 (28.7)	149 (47.5)	
3 +	42 (26.7)	75 (23.8)	
**History of abortion**			0.252^b^
0	115 (73.2)	245 (78.0)	
1 and more	42 (26.8)	69 (22.0)	
**History of IUFD**	2 (1.3)	2 (0.6)	0.604^c^
**History of preterm labour**	39 (24.8)	6 (1.9)	< 0.001^b^
**Unwanted pregnancy**	51 (32.5)	105 (33.4)	0.917^b^
**Unwanted baby sex**	31 (19.7)	62 (19.7)	1.00^b^
**Partner Violence during pregnancy**	1 (0.6)	4 (1.3)	0.669^c^
**Emotional support**	129 (82.1)	263 (83.8)	0.860^c^
**Exercise during pregnancy**	8 (5.1)	32 (10.2)	0.268^c^
Complications during pregnancy*	65 (41.4)	91 (29.0)	**0.009**^**b**^
**Permission to move during labour**	94 (59.9)	205 (65.3)	0.265^b^
**Duration of stay in the labour room (hours)**	12.3 (13.4)	8.9 (6.2)	**0.036**^**d**^
**Augmentation**	65 (41.4)	168 (53.5)	**0.015**^**b**^
**Use of pain relief method**	49 (31.3)	139 (44.3)	**0.007**^**c**^
**Doula support**	1 (0.6)	2 (0.6)	1.00^c^
**Birth attendant**			0.911^c^
Obstetrician	0	1 (0.3)	
Obstetrician resident	130 (82.8)	255 (81.2)	
Midwifery/ midwifery student	27 (17.2)	58 (18.5)	
**Type of birth**			0.698^b^
Vaginal	71 (45.2)	146 (46.5)	
Vaginal + episiotomy	81 (51.6)	162 (51.6)	
Vaginal + tear	5 (3.2)	6 (1.9)	
**Place of birth**			1.00^b^
Teaching university	130 (82.8)	261 (83.1)	
Organizational university	27 (17.2)	53 (16.9)	
**Skin to skin contact**	99 (63.1)	293 (93.3)	** < 0.001**^**b**^
**Postpartum complication, hemorrhage**	0	5 (1.6)	0.157^c^
**ICU admission**^**e**^	4 (2.5)	7 (2.2)	1.000^c^
**NICU admission**^**f**^	87 (55.4)	25 (8.0)	** < 0.001**^**b**^
**Baby weight (g)**	2462.2 (577.5)	3279.1 (392.3)	** < 0.001**^**a**^
**Baby sex (female)**	63 (40.1)	151 (48.1)	0.116^b^
**DFS**^**g**^** (10 to 100)**	35.1 (14.5)	30.3 (14.3)	< 0.001^a^

^*^Complications: Hypertension, diabetes, thyroid disorders, cardiovascular disease, sexual transmitted disease, thrombosis. The data indicate frequency (percent). *P* < 0.05 indicates significance difference

^a^Independent-Samples T Test

^b^Chi-square

^c^Fisher’s Exact

^d^Mann-Whtiney U

^e^Intensive Care Unit

^f^Neonatal Intensive Care Unit

^g^Delivery Fear Scale

A strong and statistically significant association was found between the CEQ 2.0 and P-BESS (*r* = 0.691, *p* < 0.001). The mean (SD) of the overall score of childbirth experience according to the CEQ 2.0 in the preterm and term labour groups was 2.7 (0.5) and 3.0 (0.4), respectively, out of the attainable score of 1–4. There was a statistically significant difference between the two groups in terms of overall score, “Own capacity”, “Participation” and “Perceived safety” subscales (*p* < 0.001). The prevalence of negative childbirth experience in the preterm labour and term groups was 31.8% and 14.3%, respectively, and there was a statistically significant difference between the two groups (*p* < 0.001) (Table 3).

**Table 3:** Comparison of birth experience and fear of delivery among the mothers by preterm and term labour

Variables	Preterm (*n* = 157)		Term (*n* = 314)		*p*
	**Mean (SD)**	**Median (P25-P75)**	**Mean (SD)**	**Median (P25-P75)**	
**Birth experience’s subscales**					
Own capacity (1 to 4)	2.4 (0.6)	2.5 (2.0 – 2.8)	2.7 (0.5)	2.7 (2.2 – 3.2)	< 0.001^a^
Participation (1 to 4)	2.9 (0.6)	3.0 (2.5–3.5)	3.1 (0.5)	3.2 (2.7 – 3.5)	< 0.001^a^
Perceived safety (1 to 4)	2.6 (0.7)	2.6 (2.1 -3.1)	2.9 (0.6)	3.0 (2.5 – 3.5)	< 0.001^a^
Professional support (1 to 4)	3.1 (0.7)	3.4 (2.6 – 3.8)	3.2 (0.6)	3.4 (2.8 – 3.8)	0.057^a^
Total score (1 to 4)	2.7 (0.5)	2.8 (2.3 – 3.1)	3.0 (0.4)	3.0 (2.6 – 3.3)	< 0.001^a^
Negative Birth Experience (≤ 2.5)	50 (31.8%)		45 (14.3%)		< 0.001^b^
**P-BESS**^**d**^					
Staff Professionalism and Empathy (7 to 35)	27.2 (5.8)	29.0 (24.0-31.0)	-	-	
Information and Explanation (7 to 35)	24.4 (5.2)	25.0 (20.0 – 28.0)	-	-	
Confidence in Staff (3 to 21)	9.7 (1.6)	10.0 (9.0 – 11.0)	-	-	
Total score (17 to 85)	63.5 (12.4)	66.0 (57.0 – 72.0)	-	-	

^a^Independent-Samples T Test

^b^Chi-square

^c^P-BESS: Preterm Birth Experience and Satisfaction Birth

The mean (SD) of the overall score of childbirth experience according to the P-BESS for the women with preterm birth was 63.5 (12.4) out of the attainable score of 17 to 85. The mean (SD) of the “Staff professionalism and empathy” and “Information and explanation” subscales were 27.2 (5.8) and 24.4 (5.2), respectively, from the attainable score of 7 to 35 while the mean (SD) of the “Confidence in staff” subscale was 9.7 (1.6) out of the attainable score of 3 to 21 (Table 3).

The results of the multivariable general linear model with the adjusting of demographic and obstetric characteristics showed that there was no statistically significant difference between the two groups of mothers with term and preterm births [β (95% CI): -0.06 (-0.22 to 0.09); *p* = 0.414] in terms of childbirth experience. However, the fear of delivery had a significant relationship with the childbirth experience such that as the fear of birth increased, the score of childbirth experience decreased [β (95% CI): -0.02 (-0.03 to -0.01); *p* < 0.001] (Table 4).

**Table 4:** Comparison of birth experience among the mothers by preterm and term labour according to adjusted general linear model

Characteristic	β (95% CI)	*p*^a^
**Group (Reference: Term)**		
Preterm	-0.06 (-0.22 to 0.09)	0.414
**Maternal educational (Ref: Academic)**		
Illiterate or elementary	0.01 (-0.16 to 0.19)	0.855
Secondary or high school	0.03 (-0.11 to 0.19)	0.647
Diploma	0.04 (-0.10 to 0.19)	0.574
**Husband educational (Ref: Academic)**		
Illiterate or elementary	0.06 (-0.12 to 0.25)	0.485
Secondary or high school	0.06 (-0.10 to 0.22)	0.459
Diploma	-0.03 (-0.19 to 0.12)	0.661
**Maternal Occupation (Ref: Employee)**		
Housewife	-0.24 (-0.60 to 0.11)	0.180
**Husband Occupation (Ref: Self-employed)**		
Employee	-0.08 (-0.32 to 0.14)	0.464
Labor	0.04 (-0.10 to 0.18)	0.559
**Parity (Ref: 3 and more)**		
1	-0.02 (-0.22 to 0.17)	0.813
2	0.04 (-0.14 to 0.23)	0.654
**History of preterm (Ref: Yes)**		
No	0.05 (-0.17 to 0.19)	0.933
**Complication (Ref: Yes)**		
No	-0.00 (-0.09 to 0.09)	0.944
**Augmentation (Ref: Yes)**		
No	0.13 (-0.09 to 0.35)	0.250
**Pin relief (Ref: Yes)**		
No	0.01 (-0.07 to 0.11)	0.719
**Skin to skin (Ref: Yes)**		
No	-0.05 (-0.20 to 0.09)	0.464
**NICU admission**^**b**^	-0.05 (-0.19 to 0.09)	0.493
**Gestational age (Weeks)**	0.01 (-0.01 to 0.05)	0.284
**Duration of stay in the labour room (hours)**	-0.00 (-0.01 to 0.00)	0.315
**Baby weight (g)**	-3.56 (0.00 to 7.77)	0.537
**Fear of delivery**	-0.02 (-0.03 to -0.01)	** < 0.001**

Analysis based on General Linear Model

^a^Adjusted R Squared = 0.427

^b^Neonatal Intensive Care Unit

## Discussion

To our knowledge, this is the first study to compare women's childbirth experience with preterm and term labours. The mean of the overall score of childbirth experience based on the CEQ 2.0 in preterm group (mean = 2.7) was lower than that in the term group (mean = 3.0). However, the results of the multivariate general linear model, after adjusting the demographic and obstetric characteristics, showed that there was no statistically significant difference between the two groups of mothers with preterm and term labour in terms of childbirth experience. Among the factors included in the general linear model, fear of delivery seemed to be negatively correlated with childbirth experience- the higher the fear of delivery, the lower the score of childbirth experience dropped.

Fear of delivery during labour was a predictive factor of childbirth experience. In a previous study conducted in Iran, fear of delivery was also reported as one of the predictors of low birth satisfaction among term women [32]. Fear of delivery is a negative feeling towards birth that prevents normal psychological preparation for birth [33]. During preterm labour, mothers express "fear of losing the baby" due to the early onset of labour. Studies have focused on the relationship between the severity of grief and gestational age [34, 35]. "Fear of losing a neonate" may be experienced as a "threat" in a psychological sense [36]. This feeling of threat can make a woman perceive birth as a traumatizing experience.

There was a strong and statistically significant association between the CEQ 2.0 and P-BESS. The mean overall P-BESS score was found to be 63.5 out of the maximum obtainable score of 85, which is almost similar to that reported for the UK (69.5 out of 85) [28] and Spain (84.0 out of 95) [37].

The results of multivariate general linear model showed that there was no statistically significant difference between the two groups of women with preterm and term labour in terms of childbirth experience. The results of this study are somewhat consistent with a study conducted in the Spanish context. These women expressed high satisfaction with maternity care during preterm birth. Women in Iran have also been reported to be satisfied with preterm birth [23], their experience had not been compared with women who gave birth at term, however. A qualitative study was conducted 72 h after birth on 150 mothers who had full-term, preterm and very preterm babies in Portugal [38]. The experience of mothers in relation to pregnancy, birth and interaction with the baby and their expectations in the future were investigated. Mothers with full-term babies remarked that they did not expect any problems in the care of the baby. Mothers of preterm infants, while optimistic about their competence to care for the infant, expressed fear due to the unexpected occurrence of preterm birth and the risks associated with it. Mothers of very preterm infants expressed more distress and negative emotions after birth [38]. The results of the above-mentioned study are somewhat different from ours; however, this could be due to the difference in the study design and research environment.

Some probable reasons for failing to find significant differences between preterm and term groups’ childbirth experience may include the following: 1) Certainly, women in the preterm group may experience higher fear and stress due to the unexpected onset of labour [39]. However, they also tend to undergo fewer unnecessary interventions during labour and birth than the term group, and the reduction of unnecessary interventions (such as frequent vaginal examinations, pressure on the fundal) can be a significant factor on childbirth experience [23]. The frequency of vaginal examinations in Iran's teaching centers is much more than the standard. However, in the case of preterm labour, these examinations are limited due to the possibility of infection. 2) Generally, in the teaching centers, during preterm labour, women are managed only by obstetrician and midwives, with students not present, which in a way have led to increased observance of these women’s solitude and privacy - hence enhancing their satisfaction. 3) In our study, more than 50% of preterm group babies were hospitalized in NICU when their mothers were completing the questionnaire, which may have affected the results. With their baby taken care of in NICU by the staff, the women may find themselves to be more inclined to express a positive image of labour and birth experience, or be reluctant to express their real experience. It is possible that the assessment of childbirth experience, especially regarding these mothers, could be more realistic and yield more reliable results when it is done after a period of time to provide an opportunity for the women to evaluate their experiences in retrospect [27]. 4) Preterm group is more likely to have a history of infertility and assisted reproduction treatment, leading these mothers to be satisfied with their pregnancy and childbirth and love their child [38]. 5) Also, after preterm birth, mothers may be ambivalent and have mixed feelings about their birth experience. These mothers are sometimes optimistic about the future, not expecting any problems with the baby. However, some other mothers with preterm birth express distress because of the unpredictable clinical health status of their babies [40, 41]. 6) The level of stress following a traumatic event was the strongest predictor for the occurrence of birth-related PTSD. The reason why we did not find a significant difference between the preterm and term groups in our study may have to do with the lack of assessment of perceived stress after the threat of premature birth or even after the beginning of labour in the term group. It is possible that by adjusting this factor, different results could be obtained between the two groups [42].

### Strength and limitations

The comparison of birth experience between two preterm and term groups is one of the strong points of the stud while**.** Inclusion of women only from teaching hospitals and one organizational hospital is probably one of the limitations of the study. However, due to the fact that advanced NICU equipment is usually available in such hospitals and due to the lack of insurance coverage of other hospitals in the event of a newborn being admitted to the NICU, most women refer to these hospitals in situations of threat to preterm labour. However, in the case of women who had a term labour, the results may not be generalizable to all women. Completing the questionnaires within 24 h after birth can be a limitation. Because women may give more positive responses due to the presence of staff or more negative responses due to lack of physical comfort. Future studies should investigate the effect of time on how women evaluate the childbirth experience, especially in the preterm labour group. Due to the comparison of childbirth experience, the CEQ 2.0 was used in both groups, and its results may not be precise in the preterm group. However, in order to increase the accuracy of the assessment, in addition to the CEQ, a specific and standard preterm questionnaire was also used in the preterm group. Being the first study of its kind in Iran, using standard tools are among the strengths of the study. The level of stress and the personality type of women may affect the perception of the birth experience. It is suggested these factors be examined in future studies. The preterm group may have a history of infertility treatment more than the term group, and this may affect the birth experience, however it was not investigated in this study.

## Conclusion and implications for practice

Although the prevalence of negative birth experience in the preterm group was higher than that in the term group, there was no statistically significant difference in terms of women's childbirth experience during preterm and term labour. Finally, among all the factors examined in this study, the fear of delivery during labour was the predictor of childbirth experience. Healthcare providers, managers and policymakers can use the results of this study to improve the provision of services to women. Also, in order to improve women's childbirth experience, interventions should be made to reduce fear during labour.

## Data Availability

The datasets used and analysed during the current study available from the corresponding author on reasonable request.

## Abbreviations

CEQ 2.0: Childbirth Experience Questionnaire 2.0
P-BESS: Preterm Birth Experiences and Satisfaction Scale
DFS: Delivery Fear Scale
WHO: World Health Organization
NICU: Neonatal Intensive Care Unit
